# 

**Published:** 1865-02

**Authors:** 


### CONTENTS.

### ORIGINAL ESSAYS AND COMMUNICATIONS.

Pain in Dental Surgery, - - - - - - - - - - - - 39
Extracting Teeth, - - - - - - - - - - - - - - 48
The Use of the File, - - - - - - - - - - - - - 51

### PROCEEDINGS OF SOCIETIES.

Meeting of the Iowa State Dental Society, - - - - - - - - - - 54
Annual Meeting of the Michigan Dental Association, - - - - - - 65
Address before the Michigan Dental Association, - - - - - - 70

### EDITORIAL.

Dental Specialties, - - - - - - - - - - - - - 74
Annealing Lamp, - - - - - - - - - - - - - - 76
A Letter, . . - - - - - - - - - - - - - - 86
Tongue Compressor, - - - - - - - - - - - - - 77
Obituary of Dr. S. Dunham, - - - - - - - - - - - 77

